# The evolution of rectal and urinary toxicity and immune response in prostate cancer patients treated with two three-dimensional conformal radiotherapy techniques

**DOI:** 10.1186/1748-717X-6-87

**Published:** 2011-07-27

**Authors:** Jana Vranova, Stepan Vinakurau, Jan Richter, Miroslav Starec, Anna Fiserova, Jozef Rosina

**Affiliations:** 1 Department of Medical Biophysics and Medical Informatics, 3rd Faculty of Medicine, Charles University, Prague, Czech Republic; 2 Department of Radiotherapy and Oncology, Motol University Hospital, Charles University, Prague, Czech Republic; 3 Department of Immunology and Gnotobiology, Institute of Microbiology, Academy of Sciences of the Czech Republic, v.v.i., Prague, Czech Republic; 4 Faculty of Biomedical Engineering, Czech Technical University in Prague, Kladno, Czech Republic

**Keywords:** 3-dimensional conformal radiotherapy (3DCRT), gastrointestinal and genitourinary toxicity, prostate cancer, NK cells, PTV parameters, pelvic bone marrow

## Abstract

**Background:**

Our research compared whole pelvic (WP) and prostate-only (PO) 3-dimensional conformal radiotherapy (3DCRT) techniques in terms of the incidence and evolution of acute and late toxicity of the rectum and urinary bladder, and identified the PTV-parameters influencing these damages and changes in antitumor immune response.

**Methods:**

We analyzed 197 prostate cancer patients undergoing 3DCRT for gastrointestinal (GI) and genitourinary (GU) toxicities, and conducted a pilot immunological study including flow cytometry and an NK cell cytotoxicity assay. Acute and late toxicities were recorded according to the RTOG and the LENT-SOMA scales, respectively. Univariate and multivariate analyses were conducted for factors associated with toxicity.

**Results:**

In the WP group, an increase of acute rectal toxicity was observed. A higher incidence of late GI/GU toxicity appeared in the PO group. Only 18 patients (WP-7.76% and PO-11.11%) suffered severe late GI toxicity, and 26 patients (WP-11.21% and PO-16.05%) severe late GU toxicity. In the majority of acute toxicity suffering patients, the diminution of late GI/GU toxicity to grade 1 or to no toxicity after radiotherapy was observed. The 3DCRT technique itself, patient age, T stage of TNM classification, surgical intervention, and some dose-volume parameters emerged as important factors in the probability of developing acute and late GI/GU toxicity. The proportion and differentiation of NK cells positively correlated during 3DCRT and negatively so after its completion with dose-volumes of the rectum and urinary bladder. T and NKT cells were down-regulated throughout the whole period. We found a negative correlation between leukocyte numbers and bone marrow irradiated by 44-54 Gy and a positive one for NK cell proportion and doses of 5-25 Gy. The acute GU, late GU, and GI toxicities up-regulated the T cell (CTL) numbers and NK cytotoxicity.

**Conclusion:**

Our study demonstrates the association of acute and late damage of the urinary bladder and rectum, with clinical and treatment related factors. The 3DCRT itself does not induce the late GI or GU toxicity and rather reduces the risk of transition from acute to late toxicity. We have described for the first time the correlation between organs at risk, dose-volume parameters, and the immunological profile.

## Background

Quality of life is becoming one of the most significant issues in treatment decision-making, in general, and more so in prostate cancer [1]. Thus late rectal and urinary damage became a major concern in prostate cancer; and many studies have been dedicated to the search for correlations between dose-volume, treatment-related factors, and late GI and GU toxicities [2-7]. Three-dimensional conformal radiotherapy (3DCRT) represents one of the standard treatments of prostate cancer allowing the delivery of highly "conformed" (focused) radiation to the cancer cells, while significantly reducing the amount of radiation received by surrounding healthy tissue. 3DCRT should increase the rate of tumor control, while also decreasing side effects. In spite of this focus, a higher dose to the prostate implies that the surrounding organs at risk (OARs) may also receive higher doses.

In addition, local radiation therapy (RT) alters the balance of circulating immune cells by the depletion of radiosensitive cell subsets [8]. Recently, radiation-induced functional changes in immune cells raised interest, suggesting the possible use of radiation as an antitumor immune response enhancer. Irradiation can induce leukopenia due to apoptosis of various leukocyte subpopulations. The acute exposure to low- and high-dose irradiation in mouse models changes the quantitative and functional parameters of immune cells, due to different sensitivity of splenocyte subsets to radiation doses [9]. Similar effect was described *in vitro *for cervical cancer patients [10]. Tabi *et al*. reported a prevalent loss of naive and early memory cells vs. more differentiated T cells in peripheral blood of patients during RT to the pelvis [11]. The release of the heat shock protein 72 (HSP72) during RT increased the cytotoxic CTL and NK cells [12]. Some pathological changes can be caused by the apoptosis of bone marrow (BM) stem cells and BM stromal damage [13]. Radiation-induced BM injury depends on both the radiation dose and the volume of BM irradiated [14].

We performed a prospective 4-year study, enrolling prostate cancer patients to elucidate whether the risk level of acute and particularly late rectal and urinary toxicities caused by 3DCRT techniques (whole pelvic (WP) and prostate-only (PO)), are at an acceptable level. This study reports our 42-month follow-up results, and evaluates the relationships between pretreatment, acute and late rectal and urinary syndromes and tumor-, patient- and treatment-related factors. In the last 3 years of the study, we investigated the influence of 3DCRT techniques, as well as the GI and GU toxicity on selected patient immune parameters, with special regard to the cells involved in antitumor immunity (natural killer-NK, NKT, and T).

## Methods

### Patients and clinical protocol

Data for the study were collected from 245 consecutive patients with Stage T1 to T3 clinically localized prostate adenocarcinoma, treated with 3DCRT (2004-2009) at the Department of Radiotherapy and Oncology, Motol University Hospital, Prague, Czech Republic. 48 patients with follow-up shorter than 24 months were excluded from the study. The study population thus consisted of 197 patients. Patients according to their health and lymph nodal status (classified by Prostate cancer staging nomograms-Partin tables) [15] were divided into two groups: those who underwent whole pelvic (WP) radiotherapy-irradiation of prostate, seminal vesicles, and lymph nodes followed by a prostate boost (116 patients, 59%); and prostate-only (PO) radiotherapy-irradiation of prostate and seminal vesicles (81 patients, 41%). Follow-up evaluations after treatment were performed at 3 to 6 month intervals. The median follow-up was 42 months, ranging from 24 to 55 months. Main patient characteristics and main disorders are summarized in Table 1.

**Table 1:** Patient characteristics

Characteristics	WP (n = 116)	PO (n = 81)
Age		
Median	73	74
Range	57-100	57-92
Mean ± SD	72.93 ± 8.55	74.88 ± 7.79
TNM Stage		
T0	1 (0.86%)	-
T1	6 (5.17%)	22 (27.16%)
T2	34 (29.31%)	30 (37.04%)
T3	62 (53.44%)	15 (18.52%)
T4	4 (3.45%)	1 (1.24%)
Metastases	2 (1.72%)	-
Gleason score		
Median	7	5
Range	2-9	3-10
Initial PSA [ng/mL]		
Median	19	10
Range	2-133	1-97
Mean ± SD	31.00 ± 8.67	12.46 ± 2.34
ADT	93 (80.07%)	33 (40.74%)
Previous surgery		
RP	23 (19.83%)	22 (27.16%)
TURP	7 (6.03%)	5 (6.17%)
Therapy duration (m)		
Median	57	54
Range	33-81	22-80
Mean ± SD	57.50 ± 5.56	54.04 ± 7.03
Recurrence Risk*		
Low	1 (0.86%)	19 (23.46%)
Intermediate	20 (17.24%)	38 (46.91%)
High	94 (81.03%)	23 (28.40%)
Prescription dose (Gy)		
≤ 71	60 (51.72%)	6 (7.41%)
72, 73	53 (45.69%)	72 (88.89%)
≥ 74	3 (2.59%)	3 (3.70%)
Disorders		
Without complications	49 (42.24%)	37 (45.86%)
Cystoureteritis	16 (13.79%)	15 (18.52%)
Cystoureteritis + diarrhea	15 (12.93%)	1 (1.23%)
Proctocolitis + diarrhea	28 (24.14%)	14 (17.28%)
Unknown	8 (6.69%)	14 (17.28%)

*Recurrence risk was determined according to Canadian Consensus (Lukka 2002): low risk (T1-2a, Gleason ≤ 6, PSA < 10 ng/mL), intermediate risk (T2b-2c, Gleason = 7, PSA 10-20 ng/mL), high risk (T3-4, Gleason ≥ 8, PSA > 20 ng/mL)

Acute and late GI and GU toxicities were studied in order to identify the treatment-related, clinical and patient characteristics that correlated with the severity of complications and disorders. Acute reactions included those arising during treatment or within 90 days after RT completion. Late complications were defined as those developing more than 90 days after the last treatment. Acute and late toxicities were scored according to RTOG and LENT-SOMA morbidity scale (grades 1-5). Into the category of low toxicities were encompassed the patients without the need of pharmacological intervention (grade 1), while the serious toxicity (grade ≥ 2) was under medication. In 37 cases (WP: n = 16; PO: n = 21) the immune response before treatment, during 3DCRT (day 14), and 15-20 days after treatment completion was evaluated. The protocol was approved by the local board ethics committee; and written informed consent was obtained from all patients.

### Irradiation technique, target volume and critical normal structure definition

Treatment planning and irradiation were performed with the patients in supine position (using knee and ankle supports) with an emptied rectum and "comfortably full" bladder filling. 3D conformal treatment planning based on CT images with 5 mm thickness, involved delineation of CTVs, PTVs and organs at risk, according to ICRU 50 and 62 recommendations. The plans, using MLC to shape beams, were calculated on Eclipse treatment planning system. Box technique or four wedged field technique (two lateral and two oblique fields at angles of 90°, 270°, 30° and 330°) was used. The dose was normalized to the ICRU reference point, located in the central part of the PTV or near the central axis of the beam intersection, according to ICRU 50. Dose homogeneity was between 95% and 107% of the ICRU reference dose. Dose-volume histograms were used for evaluation of doses to target volumes and organs at risk. DRRs were generated for all treatment beams and for two extra setup beams from the anteroposterior (AP) and the lateral directions (LAT).

Before the radiotherapy, the treatment plans were simulated on a conventional simulator (Ximatron and Acuity^®^, Varian Medical Systems). The isocenter was marked on the patient's skin. Patients were irradiated on a Clinac 2100 C/D (Varian) equipped with Millenium MLC-120 with beams of 18 MV or 6 MV. The dose was delivered in daily fractions of 1.8 Gy to the pelvis and of 2 Gy to the prostate and seminal vesicles, in given period five sessions per week. In the treatment room, the patients were aligned on a carbon-fiber couch panel within their immobilization device using the skin marks. Before the therapy, patient set-up was checked using electronic portal imaging (PortalVision PV-aS500^®^). Simulator images of setup fields were used as reference images for matching with portal images. Planning target volume (PTV) of the prostate (PTV3) was the entire organ (clinical target volume of prostate-CTV3), and PTV2 was the entire prostate and seminal vesicles (CTV2). Both PTVs were enlarged by 1.5 cm margin, except for the prostate-rectum interface where a 1 cm margin was again used to decrease the risk of rectal toxicity. PTV1 in the WP Group was only the CTV of lymph nodes (LNs). LNs were defined according to RTOG recommendations (treatment of only subaortic presacral LNs, contours of common iliac vessels starting at the L5/S1 interspace, external iliac contours stopping at the top of femoral heads, and contours of obturator LNs stopping at the top of the symphysis pubis) plus a 1 cm margin.

Patients from the PO group received a dose of 60 Gy in 30 fractions to the PTV2. Then the PTV3 received the prescribed dose of 10-18 Gy in 5-9 fractions. Patients from the WP group received a dose of 45 Gy in 25 fractions to the PTV1, then a dose of 20 Gy in 10 fractions to the PTV2. Finally the PTV3 received the prescribed dose 6-10 Gy in 3-5 fractions. Dose volume histograms (DVH) were generated for all PTVs and OARs. The OARs included the bladder, rectum, bone marrow, and femoral head.

### Pelvic bone marrow definition

For each patient, the pelvic bone marrow (PBM) volume was first defined according to the method described by Mell *et al*. [16]. The external contour of the PBM was delineated on the planning CT using bone windows. Three sub sites were defined: 1) iliac BM (IBM), extending from the iliac crests to the superior border of the femoral head; 2) lower pelvis (LP), consisting of the pubes, ischia, acetabula, and proximal femora, extending from the superior border of the femoral heads to the inferior border of the ischial tuberosities; and 3) lumbosacral spine (LS), extending from the superior border of the L5 vertebral body to the coccyx, but not extending below the superior border of the femoral head. To find the association of local radiation doses and changes in the number of leukocytes among patients with different body sizes, the percentage of BM irradiated volume at different doses was used as a first approximation.

### Cell separation for immunological evaluations

Citrated blood samples from patients were separated by Ficoll-Hypaque 1,077 (Sigma-Aldrich, St. Louis, MO, USA) density centrifugation for 40 min to obtain the peripheral blood mononuclear cell (PBMC) fraction.

### Flow cytometry

The fluorochrome-conjugated antibodies CD3-Pacific Blue (UCHT1), CD4-APC-Alexa Fluor 750 (S3.5), CD8-Pacific Orange (3B5) CD19-Pacific Blue (HD37), CD20-PE-Cy7 (2H7), CD38-PerCP-Cy5.5 (HIT2), and CD56-APC (MEM-188), were obtained from Dako (Glostrup, Denmark), Exbio (Prague, Czech Republic), BD Biosciences (Franklin Lakes, NJ, USA), and e-Bioscience (San Diego, CA, USA). PBMCs (5 × 10^5 ^cells/well) were stained with the antibody mixture for 30 min on ice, washed, and measured with a Becton Dickinson LSRII instrument (BD Biosciences). We included single-stain controls for further offline compensation. Measurement and subsequent analysis was performed using FACSDiva (BD Biosciences) and TreeStar FlowJo 8 (Ashland, OR, USA) software, respectively.

### NK cell-mediated cytotoxicity

The standard ^51^Cr-release assay was performed with PBMCs from patients as effectors and the NK cell-sensitive K562 erythroleukemia cell line as targets. PBMC (1.6 × 10^5 ^cells/well) were incubated with 10^4 ^Na_2_^51^CrO_4_-labeled target cells in round-bottomed 96-well microtitre plates (NUNC) at 37°C, in a humidified atmosphere containing 5% CO_2_. NK cell activity was evaluated after 4 hr of incubation, and calculated as described previously [17].

### Statistical analysis

We investigated all GI and GU toxicities (late and acute) separately. There were only 3 cases of grade 3 acute GI toxicity, only 5 cases of grade 3 acute GU toxicity, and none of grade 4 or 5. Similar observation was made for late GI toxicity (only 5 cases of grade 3, 1 of grade 4, and no instances of grade 5) and for late GU toxicity (only 13 patients of grade 3 and none of grade 4 or 5). As a consequence, we grouped the toxicity levels of all diagnosed toxicities (acute GI, acute GU, late GI, and late GU) in two categories and analyzed the binary response. The grouping of responses considered was: high toxicity (grade 2-3) vs. low or no toxicity (grades 1 or 0).

The grouped data were analyzed using multivariate logistic regression models. The list of predictive factors was the same for acute and late toxicities; except for the addition of acute toxicity, as the next predictive factor of late OAR damage. The patient-, tumor-, and treatment-related factors were as follows: 3DCRT technique used (WP vs. PO); volumes of rectum and urinary bladder; minimum, maximum, and mean dose received by the rectum and urinary bladder (D_min_, D_max_, D_mean_); percentage of rectum and urinary bladder volume receiving 40 Gy, 50 Gy, 60 Gy, and 70 Gy, respectively; patient age; stage T of TNM classification; initial PSA; Gleason score; androgen deprivation therapy (ADT) added to RT (yes/no); surgical intervention (None/Transurethral resection/Radical prostatectomy) of the prostate (NONE/TURP/RP); occurrence of hemorrhoids (yes/no); and duration of RT (weeks). A Pearson's χ^2 ^test or, in the case of small sample size, Fisher's exact test was used to examine whether there was a statistically significant difference in the occurrence and evolution of acute and particularly late GU and GI toxicity between the two observed 3DCRT techniques.

To evaluate the association of immune response and toxicity level, the patients were divided in the group T (patients with any toxicity level-grades 1-3) and group 0, those with no toxicity (grade 0). To compare the immune parameters between these groups of patients the t-test was performed. To find the relationship between immune response in prostate cancer patients and treatment related factors, Pearson's correlation coefficients were calculated.

For statistical analysis Statsoft's STATISTICA version 9 and SPSS Statistics version 18 were used. All tests were considered to be statistically significant at the level of p < 0.05. The required sample size for all performed statistical tests was calculated using IBM SPSS SamplePower software version 3.

## Results

### Logistic regression models for GI and GU toxicities

Four logistic regression models for acute GI, acute GU, late GI, and late GU toxicity were created. All models were statistically significant and adequately interpolated the data; however in both models for late toxicities, GI and GU, a large disparity between the number of patients in groups with high toxicity vs. low or no toxicity was observed. The classification ability of all four models was very good-80.0% for acute GI toxicity, 78.9% for acute GU toxicity, 76.3% for late GI toxicity, and 76.0% for late GU toxicity. The area under the ROC curve (AUC) which determines the discrimination power of the logistic model reached the following values: 0.836 for acute GI toxicity-discrimination quality according to Tape [18], "Good"; 0.810 for acute GU toxicity-"Good"; 0.784 for late GI-"Fair"; and 0.761 for late GU toxicity-"Fair".

The significance level and odds ratio for statistically significant regression coefficients are summarized in Table 2 for acute and late GI and GU toxicity. Acute GI and GU toxicities were significantly dependent on patients' increasing age, and the chance of developing high toxicity levels greaten. For late GI and GU toxicities, the larger irradiated volume of OARs (rectum and urinary bladder) enhanced the chance of high-level toxicity occurrence. Other important predictors of acute GI toxicity were the percentage of rectum volume receiving 70 Gy (the higher the percentage of rectum, the higher the chance of high level toxicity) and the 3DCRT technique used, where the high-level toxicity developed when the WP technique was used (26.16 times greater than in the case of the PO technique). The higher T stage of TNM classification and the acute GI toxicity significantly increased the probability of late GI toxicity occurrence. The results pointed to the significant association of acute GU toxicity and the percentage of the urinary bladder receiving 50 Gy, and the association of late GU toxicity with the percentage of the urinary bladder receiving 40 Gy. Both types of urinary toxicities (acute and late) were augmented by radical prostatectomy prior to radiotherapy (NONE vs. RP) that increased the occurrence of high-level toxicity for acute and late GU toxicity 7.35 times (OR = 0.136) and 11.15 times (OR = 0.090), respectively. Another important statistically significant predictor found for late GU toxicity was the PO type of 3DCRT that evoked the development of high-level toxicity 1.72 times more (OR = 0.580) in comparison with WP technique.

**Table 2:** Logistic regression models for acute and late GI and GU toxicities.

	Acute GI toxicity				Late GI toxicity		
Variable	OR	95% CI	p	Variable	OR	95% CI	p
Age	**1.097**	**1.03-1.17**	**0.006**	Volume of rectum	**1.028**	**1.00-1.06**	**0.036**
Percentage of rectum receiving 70 Gy	**1.134**	**1.03-1.25**	**0.009**	T stage of TNM classification	**4.630**	**1.09-20.00**	**0.037**
3DCRT technique WP vs PO	**26.163**	**5.10 -134.2**	**0.000**	Acute GI Low vs High	**0.115**	**0.01-0.92**	**0.042**

	Acute GU toxicity				Late GU toxicity		
Variable	OR	95% CI	p	Variable	OR	95% CI	p

Age	1.108*	**1.02-1.20**	**0.015**	Volume of urinary bladder	**1.016**	**1.00-1.03**	**0.018**
Percentage of urinary bladder receiving 50 Gy	**1.127**	**1.01-1.25**	**0.026**	Percentage of urinary bladder receiving 40 Gy	**1.144**	**1.00-1.30**	**0.045**
Surgical intervention None vs RP	**0.161**	**0.04-0.68**	**0.013**	Surgical intervention None vs RP	**0.089**	**0.01-0.85**	**0.035**
				3DCRT technique WP vs PO	**0.580**	**0.10-1.74**	**0.029**

Odds ratios (OR), 95% Confidence Intervals (CI) and significance levels (p) of Wald chi-square statistic of patient-, tumor-, and treatment-related factors that meet statistical significance are presented

### GI and GU toxicity evolution after WP and PO 3DCRT techniques

The used 3DCRT technique was proven as an important factor influencing the development of GI and GU toxicity. Consequently, we analyzed the occurrence and evolution of late GI and GU toxicity from pretreatment symptoms through acute GI and GU toxicity in each group of patients separately. The proportion of patients suffering pretreatment GU, as well as GI pathologies, was comparable in the groups undergoing either the WP or PO 3DCRT therapy. The proportion of GU toxicity did not change significantly between the WP and PO techniques in all appearing grades (0-3). The results of toxicity dynamics are summarized in Table 3. The values of the last late GI and GU toxicity observed in patients during their last inspection are shown.

**Table 3:** Scoring of GI and GU disorders for WP and PO 3DCRT techniques.

Incidence and development of acute GI/GU toxicity from pretreatment symptoms									
		**Acute GI toxicity**				**Acute GU toxicity**			
		**WP**		**PO**		**WP**		**PO**	
Pretreatment Symptoms	Acute toxicity	n	%	n	%	n	%	n	%
0 → 0		33	28.45%	40	49.38%	43	37.07%	36	44.44%
0 → 1		14	12.07%	17	20.99%	18	15.52%	12	14.81%
0 → 2		58	50.00%	20	24.69%	25	21.55%	17	20.99%
0 → 3		1	0.86%			1	0.86%		
1 → 0				1	1.23%	13	11.21%	7	8.64%
1 → 1				2	2.47%	6	5.17%	3	3.70%
1 → 2		5	4.31%	1	1.23%	4	3.45%	3	3.70%
1 → 3		2	1.72%						
2 → 0		1	0.86%			1	0.86%	1	1.23%
2 → 1								1	1.23%
2 → 2		1	0.86%			1	0.86%		
2 → 3						2	1.72%	1	1.23%
3 → 0						1	0.86%		
3 → 1									
3 → 2		1	0.72%						
3 → 3						1	0.86%		

**Development of late GI/GU toxicity from acute GI/GU toxicity**									

		**GI toxicity**				**GU toxicity**			
		
		**WP**		**PO**		**WP**		**PO**	
Acute toxicity	Late toxicity	n	%	n	%	n	%	n	%

0 → 0		29	25.00%	34	41.98%	41	35.34%	31	38.27%
0 → 1		5	4.31%	5	6.17%	13	11.21%	8	9.88%
0 → 2				2	2.47%	1	0.86%	3	3.70%
0 → 3		1	0.86%			3	2.59%	2	2.47%
1 → 0		10	8.62%	11	13.58%	17	14.66%	9	11.11%
1 → 1		4	3.45%	7	8.64%	4	3.45%	2	2.47%
1 → 2						1	0.86%	2	2.47%
1 → 3				1	1.23%	2	1.72%	3	3.70%
2 → 0		47	40.52%	9	11.11%	18	15.52%	10	12.35%
2 → 1		9	7.76%	6	7.41%	8	6.90%	8	9.88%
2 → 2		5	4.31%	5	6.17%	3	2.59%	2	2.47%
2 → 3		2	1.72%	1	1.23%	1	0.86%		
2 → 4		1	0.86%						
3 → 0		2	1.72%						
3 → 1		1	0.86%			2	1.72%		
3 → 2						1	0.86%		
3 → 3						1	0.86%	1	1.23%

**Summary of last late GI/GU toxicities dynamics**									

						**WP**		**PO**	
**Last late GI toxicity**						n	%	n	%

Patients without toxicity						29	25.00%	34	41.98%
Decrease of toxicity (G1,2,3→G0)						59	50.86%	20	24.69%
Patients with moderate toxicity-G1									
Development G0 → G1						5	4.31%	5	6.17%
Unchanged grade of toxicity G1						4	3.45%	7	8.64%
Decrease of toxicity from G2, 3→ G1						10	8.62%	6	7.41%
Patients with high level toxicity G2, 3, 4						9	7.76%	9	11.11%

						**WP**		**PO**	
**Last late GU toxicity**						n	%	n	%

Patients without toxicity						41	35.34%	31	38.27%
Decrease of toxicity (G1,2,3 → G0)						35	30.17%	19	23.46%
Patients with moderate toxicity-G1									
Development G0 → G1						13	11.21%	8	9.88%
Unchanged grade of toxicity G1						4	3.45%	2	2.47%
Decrease of toxicity from G2, 3→G1						10	8.62%	8	9.88%
Patients with high level toxicity G2, 3, 4						13	11.21%	13	16.05%

In the cohort of patients included in the WP group, pretreatment GI toxicity of grade 2 was found in the history of 2 patients (1.72%), and only 1 patient (0.86%) showed grade 3. During treatment or within the first 90 days after treatment, acute grade 2 GI toxicity occurred in 65 (56.03%) and grade 3 GI toxicity in 3 patients (2.59%). The severe late GI toxicity of grade 2 occurred in 5 (4.31%), grade 3 in 3 patients (2.59%), and grade 4 in 1 patient (0.86%). There were no late grade 5 GI toxicity-suffering patients in this group. Pretreatment GU damage of grade 2 was found in the history of 4 patients (3.44%) and grade 3 in the history of 2 patients (1.72%). WP 3DCRT evoked acute grade 2 GU toxicity in 30 (37.04%) and acute grade 3 GU toxicity in 4 patients (3.45%). Severe late GU toxicity of grade 2 occurred in 8 patients (5.76%) and grade 3 in 6 patients (7.41%). There were no late grade 4 or 5 GU toxicities observed.

**Figure 1:**
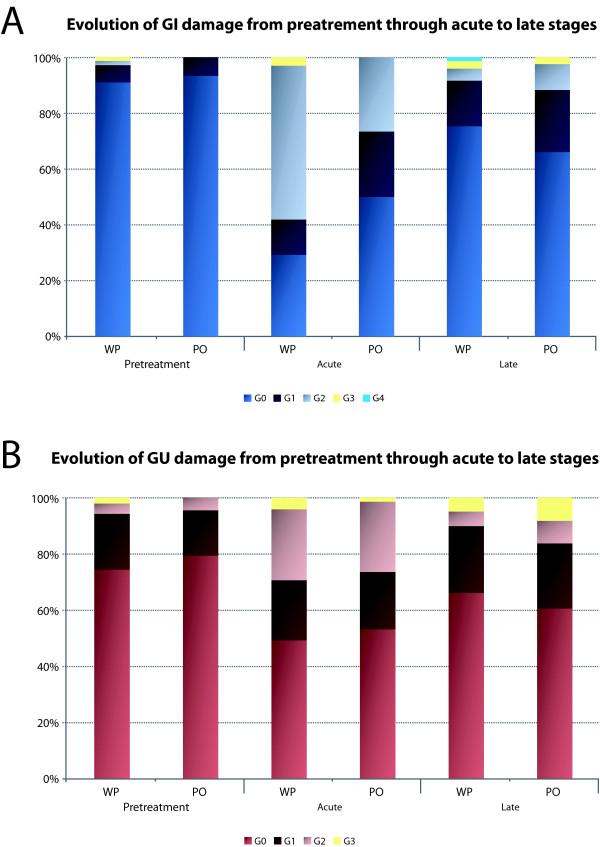
**Summary of GI and GU symptoms scoring before and after 3DCRT**. Comparison of GI (A) and GU (B) toxicity between the PO (n = 106) and the WP (n = 139) patient groups. Patients were scored according to the modification of RTOG morbidity scale. Percentage of occurrence of grades G0, G1, G2, and G3 of pretreatment pathology, acute, and late GU and GI toxicities are demonstrated.

None of the patients in the PO group suffered grade 2, 3 or 4 pretreatment GI disorders. During RT or within the first 90 days after PO 3DCRT, acute grade 2 GI toxicity occurred in 21 cases (25.93%), and there were no patients with grade 3 or 4 GI toxicity. 7 patients (8.64%) suffered severe late grade 2 GI toxicity, and 1 patient (1.23%) grade 3. Prior to radiotherapy, 3 patients (3.77%) had grade 2 toxicity, and none had grade 3 GU toxicity. Acute grade 2 GU toxicity developed in 20 (24.69%) and grade 3 in 1 (1.23%) patients. Late grade 2 GU toxicity occurred in 7 (8.64%) and grade 3 in 6 (7.41%) patients. None of the patients in the cohort had grade 4 of GU toxicity. Figure 1 summarizes the proportion of evolution of GI (Figure 1A) and GU (Figure 1B) toxicity events from pretreatment through acute to late damage, for both the WP and PO patient groups. The only disparity between the two 3DCRT techniques was found in the case of development of acute GI toxicity, where a large increase of high level toxicity grades ≥ 2 was observed in the WP group compared to the PO group. On the other hand, results from Table 3 illustrate the diminution of toxicity from grades 1-3 to no toxicity (grade 0), more prominent in the WP group relative to the PO group. The Pearson's χ^2 ^test was performed to determine the statistical significant difference between the WP and PO 3DCRT techniques, which was observed only in the occurrence of acute GI toxicity (p = 0.0001).

### Correlation between the 3DCRT parameters, GI/GU toxicity and immune response

We screened the immunological parameters, number of leukocytes, distribution of lymphocyte populations (T, B, NK, and NKT cells) and their subsets in the peripheral blood of patients before, throughout and after the finishing of 3DCRT, and correlated them to dose volume parameters, as well as to the volume of irradiated bone marrow.

**Figure 2:**
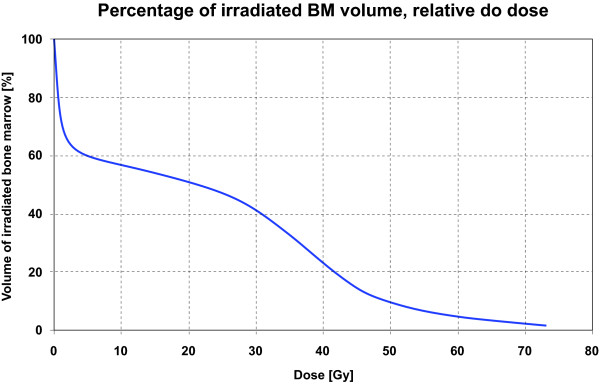
**Relationship between the percentage of irradiated volume of bone marrow and the dose applied**.

**Figure 3:**
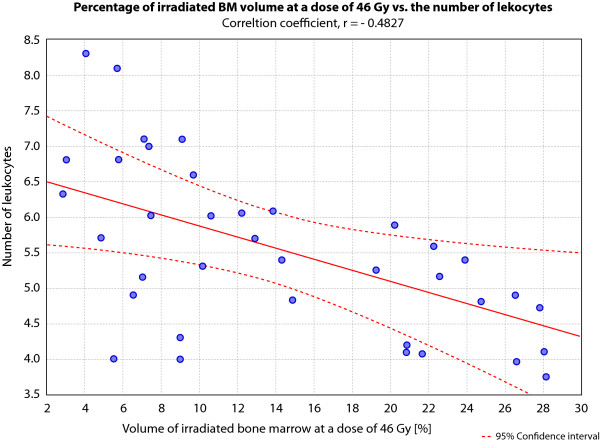
**Scatter plot showing the correlation between the percentage of irradiated volume of bone marrow and the decrease of number of leukocytes**.

The relationship of the applied dose and the percentage of volume of bone marrow irradiated are presented in Figure 2. The highest correlation occurred at a dose of 46 Gy, as depicted in Figure 3. We found that the bone marrow irradiation had a significant negative association with the number of leukocytes, but did not influence the proportion of NK cells during the irradiation in doses ranging from 44 Gy to 54 Gy (Table 4). Doses lower than 44 Gy and higher than 54 Gy, did not exhibit statistically significant correlations with leukocyte number. In the scope of PBM irradiation, we found a positive correlation between low doses (1-43Gy) and NK cell numbers during RT (Table 4). Blood samples of patients receiving 34-35 Gy to the bone marrow demonstrated significantly increased proportion of NK (p = 0,002), NKT (p = 0,005) and cytotoxic T cells (p = 0,018) after the end of therapy. Moreover, T lymphocyte proportions in the patient's blood correlated positively with the higher doses (47-62 Gy) of irradiated PBM.

**Table 4:** Pearson's correlation coefficients between bone marrow irradiation and immune parameters.

Dose [Gy]	Volume [%]		Number of leukocytes		Proportion of NK cells	
	Median	Range	Correlation coefficient	p	Correlation coefficient	p
5	44.54	30.31-98	-0.3177	0.140	**0,5185**	**0,019**
6	43.92	29.57-98	-0.3161	0.142	**0,5197**	**0,019**
7	43.38	28.95-98	-0.3161	0.142	**0,5225**	**0,018**
8	42.77	28.42-98	-0.3162	0.142	**0,5239**	**0,018**
9	42.31	27.95-97	-0.3170	0.141	**0,5236**	**0,018**
10	41.86	27.53-97	-0.3188	0.138	**0,5224**	**0,018**
11	41.34	27.12-97	-0.3213	0.135	**0,5261**	**0,018**
12	40.74	26.74-96	-0.3256	0.129	**0,5196**	**0,019**
13	40.13	26.36-96	-0.3314	0.122	**0,516**	**0,020**
14	39.63	26.00-96	-0.3361	0.117	**0,5147**	**0,020**
15	39.13	25.66-95	-0.3390	0.114	**0,5133**	**0,021**
16	38.66	25.34-95	-0.3402	0.112	**0,5124**	**0,021**
17	38.20	25.03-95	-0.3411	0.111	**0,5117**	**0,021**
18	37.77	24.72-94	-0.3423	0.110	**0,5107**	**0,021**
19	37.19	24.40-94	-0.3446	0.107	**0,5096**	**0,022**
20	36.35	24.05-94	-0.3463	0.105	**0,5083**	**0,022**
21	35.70	23.70-93	-0.3481	0.104	**0,5065**	**0,023**
22	35.20	23.33-93	-0.3496	0.102	**0,5036**	**0,024**
23	34.66	22.91-92	-0.3517	0.100	**0,4984**	**0,025**
24	34.13	22.37-91	-0.3675	0.084	**0,4771**	**0,033**
25	33.53	21.61-83	-0.3713	0.081	**0,4579**	**0,042**
**44**	**10.97**†	**4.38-38.66**	**-0.4619**	**0.027**	0,4270	0,060
**45**	**9.97**	**4.22-35.05**	**-0.4645**	**0.026**	0,3986	0,082
**46**	**9.08**	**4.07-28.04**	**-0.4827**	**0.020**	0,4153	0,069
**47**	**8.39**	**3.93-23.31**	**-0.4769**	**0.021**	0,3906	0,089
**48**	**7.70**	**3.81-21.61**	**-0.4731**	**0.023**	0,3935	0,086
**49**	**7.07**	**3.50-20.48**	**-0.4701**	**0.024**	0,4023	0,079
**50**	**6.54**	**3.15-19.58**	**-0.4710**	**0.023**	0,4130	0,070
**51**	**6.00**	**2.83-18.84**	**-0.4751**	**0.022**	0,4178	0,067
**52**	**5.55**	**2.55-18.16**	**-0.4747**	**0.022**	0,4187	0,066
**53**	**5.21**	**2.30-17.50**	**-0.4709**	**0.023**	0,4201	0,065
**54**	**4.98**	**1.95-16.82**	**-0.4655**	**0.025**	0,4208	0,065

The number of leukocytes and NK cell percentages were correlated to dose received and volume of irradiated bone marrow (n = 37)

*Required sample size for the obtained correlation coefficients (for α = 0.05 and power of the test β = 0.80) was calculated 32-34 patients

†Statistically significant results are marked in bold

Increased number of resting and terminally differentiated NK cells correlated with several dosimetric parameters, and GI and GU toxicity. Table 5 summarizes the Pearson's correlations between the immune and dosimetric variables on day 14 of RT, and 15-20 days post-radiotherapy. Negative correlation throughout the RT was detected between the NKT cell and T lymphocyte proportion and the volume of the rectum receiving lower and higher doses, respectively. After completion of RT the NK and NKT cells were found to be more sensitive to higher doses. However, positive correlation was found between differentiating B lymphocytes, and the irradiated volume of rectum and bladder receiving 70 Gy.

**Table 5:** Pearson's correlation coefficients of immune cells proportions with dosimetric parameters

14^th ^date of 3D CRT				15-20 days after completion of 3D CRT			
**Variable vs**.	Variable	Pearson's correlation	p	**Variable vs**.	Variable	Pearson's correlation	p
T cells (CD3+CD56-)	D_min_	-0.5869 (20)*	0.012	NK cells (CD3-CD56low)	Percentage of rectum receiving 70 Gy	-0.5436 (23)	0.024
	D_mean_	-0.5068 (27)	0.032				
	D_max _of rectum	-0.4918 (29)	0.038				
	D_max _of urinary bladder	-0.6089 (18)	0.007				
	Percentage of urinary bladder receiving 70 Gy	-0.4906 (29)	0.007				

NKT cells (CD3+CD56+)	D_min _of rectum	-0.5776 (20)	0.012	NKT cells (CD3+CD56+)	D_max _of rectum	-0.6755 (14)	0.000
	D_mean _of rectum	-0.7243 (12)	0.001		Percentage of rectum receiving 70 Gy	-0.4148 (42)	0.031
	Percentage of rectum receiving 40 Gy	-0.7363 (11)	0.000		D_max _of urinary bladder	-0.6210 (17)	0.001
	Percentage of rectum receiving 50 Gy	-0.5613 (22)	0.015				

NK cells (CD3-D56low)	D_min _of rectum	0.3963 (47)	0.033	Activated B cells (CD19+CD20+ CD38+)	D_min _of rectum	0.4582 (34)	0.016
	D_mean _of rectum	0.3724 (53)	0.047		D_mean _of rectum	0.4342 (38)	0.024
	Percentage of urinary bladder receiving 70 Gy	0.5152 (26)	0.004		Percentage of rectum receiving 50 Gy	0.4011 (46)	0.038
					Percentage of rectum receiving 60 Gy	0.5800 (20)	0.002

Terminally differentiated NK cells (CD3-CD56+)	D_min_	0.4887 (30)	0.040	Terminally differentiated NK cells (CD3-CD56+)	D_max _of rectum	-0.5549 (22)	0.000
	Percentage of rectum receiving 70 Gy	0.4835 (30)	0.042		D_max _of urinary bladder	-0.4608 (34)	0.016
	Percentage of urinary bladder receiving 70 Gy	0.5226 (26)	0.026				
	GI, GU toxicity	0.5166 (26)	0.028				

*Required sample size for correlation coefficient for α = 0.05 and power of the test β = 0.80 is given in the brackets

**Figure 4:**
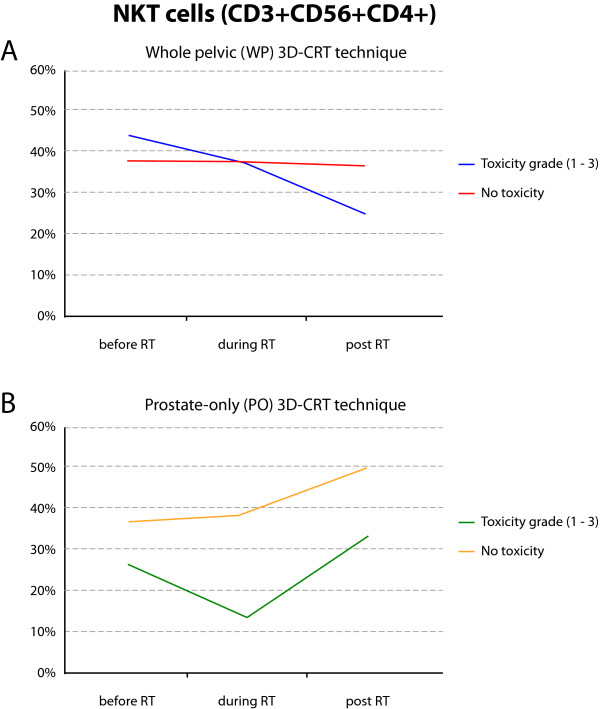
**Cumulative effect of radiation vs. toxicity on NKT cell proportions in the course of 3DCRT**. CD4+ out of CD3+CD56+ NKT cells were evaluated in the PBMC of patients suffering GI, GU toxicities of grades 1-3 (T) or without any toxicity (0) undergoing WP (n = 16; A) or PO (n = 21; B) types of radiotherapy. Pooled data of patients in each treatment group, at all time points tested, are presented as mean values, where standard deviation does not exceed 10%.

The evaluation of GI and GU toxicity effects in the WP (but not PO) group of patients revealed significant up-regulation of T lymphocyte numbers (p = 0.047) and NK cell effector function (p = 0.038) during radiotherapy, as well as in patients developing acute GU toxicity. Late GU toxicity-suffering patients had a significantly increased number of CD8+ cytotoxic T cells, (p = 0.002) and NK cell killing capability (Table 6). All statistically significant correlation coefficients met the conditions of required sample size. The GI and GU toxicity side effects (after the completion of 3DCRT), but not 3DCRT itself, significantly decreased the distribution of NKT cells in the WP group (Figure 4A). However, the patients treated with the PO 3DCRT, suffering GI and GU toxicities, had a lower number of NKT cells during the entire follow-up (Figure 4B).

**Table 6:** Influence of GI/GU toxicity on antitumor immune response.

Toxicity	Variable	Mean ± SD (T)	Mean ± SD (0)	p-value	N (T)	N (0)
**Acute GU** 14^th ^day of 3D-CRT	% of T cells (CD3+D56-)	68.41 ± 0.70	58.33 ± 8.99	0.047	26	11 (6)*
**Acute GU** 14^th ^day of 3D-CRT	Cytotoxicity	13.71 ± 5,21	6.54 ± 3.12	0.038	26	11 (6)
**Late GU** 15-20 days after 3D-CRT	% of CTL (CD3+CD8+)	15.99 ± 6.52	8.55 ± 2.26	0.002	13	24 (7)
**Late GI** 15-20 days after 3D-CRT	Cytotoxicity	25.44 ± 4.96	13.82 ± 3.68	0.032	14	23 (2)

For comparison of immune parameters between the group of patients suffering from any acute and late GU or GI toxicity (T), and the group of patients without toxicity side effects (0) after 3DCRT the t-test was applied.

*Required sample size in each group for given standard deviation and difference of means between groups for α = 0.05 and power of the test β = 0.80 is given in the brackets

## Discussion

In this study two different 3DCRT techniques (WP and PO) were analyzed and the degree of association was determined between the occurrence and evolution of acute and late GI and GU toxicities and the treatment related characteristics in patients entering our hospital. Important findings include: (i) a higher proportion of acute GI toxicity in the WP 3DCRT technique group and conversely a slightly higher proportion of late GI and GU toxicity in the PO patient group; (ii) acute GI toxicity as a significant predictor of late GI toxicity; (iii) a strong dependence of the occurrence and evolution of acute GI toxicity and of late GU toxicity on which 3DCRT technique is used; (iv) the association of both acute and late GU toxicity and radical prostatectomy performed prior to radiotherapy; (v) the influence of age on both acute GI and GU toxicities; (vi) a correlation between the percentage of volume of irradiated bone marrow and a decreased number of leukocytes; and (vii) the influence of radiotherapy preferentially on NK, NKT and T cell subpopulations.

We found an increase of acute vs. pretreatment GI symptoms predominantly in the WP group, even if the patients were irradiated with lower doses compared with the PO 3DCRT group. We assume that the limiting factor in high-volume irradiation is not the dosimetric parameters, but the overall patient tolerance. In addition, the WP technique was undergone by patients with advanced stages of disease, lower overall health status, and suppressed immune functions. These observations are supported by data of Jereczek-Fossa [19] and Schultheiss *et al*. [20]; however, some investigators didn't demonstrate this correlation [21]. On the other hand, the diminution of late GI and GU toxicities to grade 1 or to no toxicity in the majority of acute toxicity (grade 1-3) suffering patients, was observed also in the WP 3DCRT group.

Our data regarding the frequency of severe toxicities are similar to those of other series, despite the fact that a direct comparison of toxicities is difficult due to the existence of many modified versions of the classification, and modifications of grading scales. Similarities were found between our results, the RTOG 9413 [22] analysis, and the GETUG-01 [23] prospective study. The diversity in the diagnostics could be created by individual physicians due to the subjectivity of the scoring system, when the same toxicity could be graded differently. Due to the findings of decreased late GI and GU toxicities after 3DCRT in the cohort of our patients, we compared these results with the studies using hypofractionated stereotactic body radiotherapy SBRT, which is a new modality of localized prostate cancer RT. The SBRT, together with innovations in image-guidance technology, is able to automatically correct the movement of the prostate during treatment, and deliver highly-conformal beam profiles, which have greatly enhanced the capability of delivering high dose fractions to a well-defined target, with sharp dose fall-off towards the bladder and rectum. Most of the studies concerning SBRT as a monotherapy or even as a boost following external beam radiotherapy presented only negligible incidence of severe late GI and GU toxicity. Katz et al. [24,25], Freeman et al. [26] and other authors [27] reported milder toxicity profiles in comparison with our results, particularly in the case of late GI and GU impairments. On the contrary, Jabbari et al. [28] presented in their study similar results as our ones in the occurrence of severe late GU toxicity (grades 2-3), and even worse outcomes in evolution of acute GU toxicity.

The analysis of GU toxicity is difficult, due to interference with pre-existing dysfunction, age-related diseases, and previous urological surgery [21,29]. We have to remember that some of these pre-existing symptoms could have been erroneously interpreted as acute or even late GU toxicity. On the other hand, late bladder damage can occur with a long latency time, potentially resulting in the underestimation of the real severity of late toxicity [30]. The difference in the time of clinical manifestation could be the reason why some researchers demonstrated the correlation of acute and late GI, but not GU toxicity [31]. These outcomes were proved in our study, as well.

The development of acute 3DCRT- induced GI and/or GU damage was generally mild in both groups; and none of the patients had an interruption of radiotherapy due to toxicity side effects. The risk of both acute GI and GU reactions depended preferentially on the age of patients, in agreement with the results demonstrated by Jereczek-Fossa *et al*. [31]. The biological variables and different clinical decisions based on patient age could participate on the final outcome. The association of acute GU toxicity with the percentage of the urinary bladder receiving 50 Gy found in our study was in accordance with the results of Fiorino *et al*.[32] and other authors [21,29], who reported a significant correlation between DVH parameters and incontinence. Similarly, the acute GI toxicity associates with the percentage of rectum receiving 70 Gy as demonstrated also by the Italian Association for Radiation Oncology (AIRO) Group on Prostate Cancer (AIROPROS) 0101 trial (previous retrospective investigation [5], who described that the dose of 70 Gy at rectum was predictive for late G2-G3 bleeding), AIROPROS 0102 [33], and by others [34,30,7,35]. Both late GI and GU toxicities positively associated with the volume of the irradiated organ at risk, rectum and urinary bladder, respectively. Furthermore, the late GI toxicity is associated with stage T of TNM classification of the disease, and is strongly influenced by acute GI toxicity. These finding are in agreement with the published data of Heemsbergen *et al*. [36]. The risk of late GU reactions depended on the percentage of urinary bladder volume receiving 40 Gy, the 3DCRT technique used, and the previous urological surgery [21,29].

Originally, the primary mechanism of RT in cancer reduction has been considered the neoplastic cell DNA damage. However, Takeshima *et al*. have found that tumor-specific CTL, which were induced in the draining lymph nodes and tumor tissue of mice by RT, are fundamental to the inhibition of cancer growth [37]. The immunological evaluation performed during 3DCRT showed a positive correlation of the number of activated NK cells and the proportion of terminally differentiated tumor targeted cytotoxic effectors with GI and GU toxicities. Both of these subpopulations returned to normal values or decreased after completing RT. In contrast, T lymphocytes were decreased during RT and normalized after its completion; while NKT cells were down-regulated in all time periods. The acute GU and late GI and GU toxicities significantly increased the T cell proportion, NK cell-mediated cytotoxicity, and cytotoxic T cell numbers. We assume that these changes are caused by stress conditions induced by RT-damaged and GI or GU toxicity-affected tissues, eliciting stimulation of cytotoxic cells (NK and CTLs). These RT effects could be due to inflammation following increased apoptotic/necrotic events in the involved tissues. The surface expression or extracellular release of stress proteins (e.g. MICs, Hsp70), following tumor cell damage by RT, can play a key role in immune system modulation [38]. These molecules are ligands of the NK cell activation receptor NKG2D [39], and can stimulate NK cell functional maturation. Particularly, Hsp72 can act as an immunological adjuvant [39,40], participating in the non-self recognition of prostate cancer cells. Thus we can hypothesize, according to results of Hurwitz *et al*. [12] that the enhanced immune function, involving resting and terminally differentiated NK cells during 3DCRT, as well as the up-regulation of CTL number and the NK cell-mediated cytotoxicity in GI or GU suffering patients, could follow the release of HSPs either evoked by radiation or by GI or GU toxicity-induced cellular stress.

## Conclusion

Our RT series included 197 patients who were treated in one center and may serve as a basis for comparison with other oncology centers, particularly in the Czech Republic. We found a strong dependence of the development of GI and GU disorders on the 3DCRT technique applied. Most important from a clinical point of view and the overall quality of life of prostate cancer patients after 3DCRT treatment was the diminution of late GI and GU toxicity to grades 0-1 in a majority of acute toxicities of patients suffering grades 1-3. The relevance of our study lies in the complex evaluation of clinical and radio-therapeutical variables describing the correlations between OARs parameters, GI and GU toxicity, phenotype, and the functional profile of immune cells. Our results brought a new insight into the 3DCRT impact on OARs and the antitumor immune response.

## List of abbreviations

3DCRT: three-dimensional conformal radiotherapy; ADT: androgen deprivation therapy; AUC: area under the curve; BM: bone marrow; CD: cluster of differentiation; CT: computer tomography; CTL: cytotoxic T lymphocyte; CTV: clinical target volume; DVH: dose volume histogram; GI: gastrointestinal; GU: genitourinary; IBM: iliac bone marrow; ICRU: International Commission on Radiation Units; LENT-SOMA: Late effects in Normal Tissues Subjective, Objective, Management and Analytical scales; LN: lymph nodes; LP: lower pelvis; LS: lumbosacral spine; MLC: multileaf collimator; NK: natural killer; OAR: organs at risk; OR: odds ratio; PBM: pelvic bone marrow; PBMC: peripheral blood mononuclear cells; PO: prostate-only; PSA: prostate-specific antigen; PTV: planning target volume; ROC: receiver operating characteristic; RP/TURP: radical prostatectomy/transurethral resection; RT: radiotherapy; RTOG: Radiation Therapy Oncology Group; WP: whole pelvic.

## Competing interests

We have no personal or financial conflicts of interest, and have not entered into any agreement that could interfere with our access to the data on the research, or upon our ability to analyze the data independently, to prepare manuscripts, and to publish them.

## Authors' contributions

All authors have read and approved the final manuscript. JV, JRo and AF prepared the design of the manuscript; SV made the treatment planning and selected the patients; MS managed the experimental schedule and collected the samples; JRi and AF completed the immunological results; JV created the statistical evaluations; and JV together with AF, SV, and JRi wrote the paper.
